# Purification and Characterization of Cytoplasmic NADP^+^-Isocitrate Dehydrogenase, and Amplification of the *Nadp*^+^-*IDH* Gene from the Wing-Dimorphic Sand Field Cricket, *Gryllus firmus*

**DOI:** 10.1673/031.011.5301

**Published:** 2011-04-18

**Authors:** Anthony J. Zera, Susan Newman, David Berkheim, Christine Black, Lindsay Klug, Erica Crone

**Affiliations:** School of Biological Sciences, University of Nebraska, Lincoln, NE 68588 USA

**Keywords:** cricket, enzyme, evolution, lipid biosynthesis

## Abstract

Cytoplasmic NADP^+^-isocitrate dehydrogenase (NADP^+^-IDH) has been purified and characterized, and its gene sequenced in many animal, plant, and yeast species. However, much less information is available on this enzyme-gene in insects. As a first step in investigating the biochemical and molecular mechanisms by which NADP^+^-IDH contributes to adaptations for flight vs. reproduction in insects, the enzyme was purified to homogeneity in the wing-dimorphic cricket, *Gryllus firmus*, characterized, and its corresponding gene sequenced. Using a combination of polyethylene glycol precipitation, Cibacron-Blue affinity chromatography, and hydrophobic interaction chromatography the enzyme was purified 291-fold (7% yield; specific activity = 15.8 µmol NADPH/min/mg protein). The purified enzyme exhibited a single band on SDS PAGE (46.3 kD), but consisted of two N-terminal amino acid sequences that differed in the first two amino acids. Purified enzyme exhibited standard Michaelis-Menten kinetics at pH 8.0 and 28° C (K_M(NADP+)_ = 2.3 ± 0.4 µM; K_M(Na+-Isocitrate)_ = 14.7 + 1.8 µM). Subunit molecular mass and K_M_S were similar to published values for NADP^+^-IDHs from a variety of vertebrate and two insect species. PCR amplification of an internal sequence using genomic DNA followed by 3′ and 5′ RACE yielded a nucleotide sequence of the mature protein and translated amino-acid sequences that exhibited high similarity (40–50% and 70–80%, respectively) to sequences from insect and vertebrate NADP^+^-IDHs. Two potential ATG start codons were identified. Both Nterminal amino-acid sequences matched the nucleotide sequence, consistent with both enzyme forms being transcribed from the same gene, although these variants could also be encoded by different genes. Bioinformatic analyses and differential centrifugation indicated that the majority, if not all, of the enzyme is cytoplasmic. The enzyme exhibited high specific activity in fat body, head and gut, and a single band on native PAGE.

## Introduction

NADP^+^-dependent isocitrate dehydrogenase (NADP^+^-IDH, EC 1.1.1.42) catalyzes the oxidative decarboxylation of isocitrate to aoxoglutarate with the concomitant production of NADPH. Eukaryotic NADP^+^-IDH plays several important physiological roles that have been studied most intensively in mammals, plants, and fungi (e.g. *Saccharomyces cerevisiae*) (Plaut et al. 1983; Gonzalez-Villasoñor and Powers 1985; Loftus et al. 1994; Corpas et al. 1999; Koh et al. 2004; Contreras-Shannon and McAlister-Henn 2004; Liu et al. 2006). In the cytoplasm, NADP^+^-IDH often contributes significantly to the NADPH pool required for reductive fattyacid biosynthesis, while in the mitochondrion this enzyme provides NADPH for maintenance of proper oxidation-reduction balance and protection against oxidative damage. Finally, within peroxisomes, NADP^+^-IDH is involved in the reduction of unsaturated fatty acids as a prelude to βoxidation. NADP^+^-IDH isozymes have been purified and characterized, and the genes encoding them have been sequenced in a variety of vertebrates, plants, and fungi (Gonzalez-Villaseñor and Powers 1985; Huh et al. 1993; Loftus et al. 1994; Szewczyk et al. 2001; Contreras-Shannon and McAlister-Henn 2004).

NADP^+^-IDH has been much less studied in insects. To our knowledge, cytoplasmic NADP^+^-IDH has thus far been purified to homogeneity and characterized in only two insect species several decades ago: the fruitfly *Drosophila melanogaster* (Williamson et al. 1980) and the silkworm, *Bombyx mori* (Miake et al. 1977a). As in other eukaryotes, this enzyme appears to play an important role in producing NADPH for fatty-acid biosynthesis. For example, using null or leaky mutants, Geer et al. (1979) estimated that NADP^+^-IDH produces about 22% of the total NADPH pool in *D. melanogaster*. The *Nadp-Idh* nucleotide and translated amino acid sequence have also been reported for these two insect species (see Discussion).

NADP^+^-IDH has recently become a focus of study in the wing-polymorphic cricket *Gryllus firmus* in the context of the biochemical basis of adaptations for flight versus reproduction (Zhao and Zera 2002; Zera and Zhao 2003; Zera 2005; Zera and Harshman 2011). Wing polymorphism is an ecologically-important adaptation found in natural populations of many insect species (2005). The polymorphism consists of a flight-capable morph with fully developed wings and flight muscles (LW = long-winged) and a flightless morph with vestigial wings and vestigial flight muscles (SW = short winged). Importantly, the flightless SW morph exhibits substantially enhanced egg production, relative to the LW morph, and thus trade-offs flight capability for fecundity. An important biochemical component of this trade-off is the reduced accumulation of triglyceride flight fuel by the SW morph, which allows increased nutrient allocation to egg production. Rates of fatty acid and triglyceride (flight fuel) biosynthesis during adulthood were substantially reduced in the flightless-reproductive morph of *G. firmus*, compared with its flight-capable counterpart, as were specific activities of many cytosolic, lipogenic enzymes including cytoplasmic NADP^+^-IDH (Zhao and Zera 2002; Zera and Zhao 2003; Zera 2005).

A goal of the research presented here on the biochemical basis of the dispersal-reproduction trade-off is to identify the mechanisms responsible for the reduced activities of lipogenic enzymes of the fat body in flightless vs. dispersing *G. firmus*. As a first step, NADP^+^-IDH from *G. firmus* was purified to homogeneity, and the enzyme was characterized with respect to important structural, physical, and kinetic properties. In addition, the NADP^+^-IDH gene from *G. firmus* that encodes this enzyme was amplified and sequenced using cDNA as well as genomic DNA.

**Figure 1.**
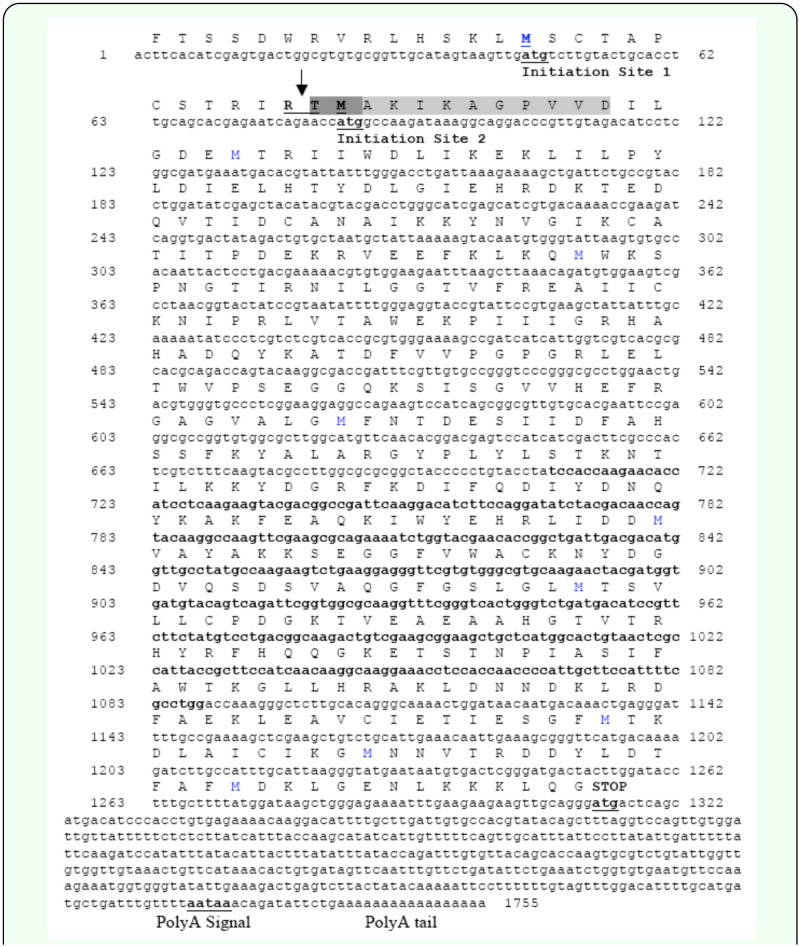
Nucleotide and translated amino-acid sequences of the *Nadp*^+^-*isocitrate dehydrogenase* gene amplified from *Gryllus firmus*. The two presumptive start codons at bp 45–47, and 84–86, the stop codon, and polyA signal are in bold and underlined. The 381 bp internal sequence (bp 708–1088) that was initially amplified using degenerate primers is in bold. The light shaded (AA 29–38) and dark plus light shaded (AA 27–38) amino acid sequences are identical to those obtained from N-terminal aminoacid sequence analysis of the purified NADP^+^-IDH protein (see Table 2). The underlined two amino acids “RT” (AA 26–27) denote the presumptive tryptic cleavage site that potentially gave rise to the observed N-terminal sequence starting at Thr(27) (IDH-2(tr), Table 2) (see Results and Discussion). High quality figures are available online.

## Materials and Methods

### Maintenance of stocks

*Gryllus firmus* used in the present study were taken from standard long-winged (LW) and short-winged (SW) artificially-selected lines that were produced from a population of *G. firmus* founded from field-collected crickets. These are the same stocks that had been used in previous studies of lipid metabolism of this species (Zera 2005). Selected lines were maintained as described previously (e.g. 28° C, 16:8 L:D photoperiod, fed a standard diet).

### NADP^+^-IDH gene amplification and sequencing

Genomic DNA was extracted from a single juvenile *G. firmus* using Chelex DNA extraction (as in Crone et al. 2007), while RNA was extracted from the abdomen of a single adult female using Trizol LS reagent according to the manufacturer's instructions. An internal portion of the NADP^+^-IDH gene was first amplified by PCR using genomic DNA as the template and degenerate primers constructed from conserved NADP^+^-IDH mRNA sequences of mammals (*Homo sapiens, BC021046; Bos taurus, NM_181012; Rattus norvegicus, NM_031510; Mus musculus, NM_010497*) and insects (*Drosophila melanogaster, NM_168267; Bombyx mori, DQ311191; Periplanita americana, AF041471*; all sequences from NCBI database) using ClustalW (Thompson et al. 1994; see legend of Figure 1). The forward primer was “AGCACCAAIAACACIATTCTGAAG” (bp 708–731, Figure 1) while the reverse primer was “CCAGGCAAAIATIGAAGCAATGGG” (bp 1065–1088). The PCR reaction (50µl) contained 36 µl distilled water, 5 µl PCR buffer (200 mM Tris-HCl, pH 8.4; 500 mM KCl; Invitrogen), 2.5 µl DMSO, 1 µl 10mM dNTP mixture (Invitrogen, www.invitrogen.com), 1.5 µl 50 mM MgCl_2_, 1 µl each of 10 µM solutions of forward and reverse primers, 1 µl 5U/µl *Taq* DNA polymerase (Invitrogen), and 1 ul genomic DNA solution. The PCR reaction conditions were as follows: 94° C for 2 min; 35 cycles of 94° C for 30 sec, 53° for 30 sec, and 72° for 30 seconds; 72 C for 2 min; and 22° for 10 seconds. A 10 µl aliquot of the reaction mixture run on a 2% agarose gel (TAE buffer, 120 V for 30 min, stained with ethidium bromide) revealed two amplicons: one of approximately 380 bp, which was the expected size for an NADP^+^-IDH amplicon, as well as a 530 bp amplicon. The approximately 380 bp amplicon was gelpurified, reamplified by PCR under the same conditions as given above, and cloned using the pGEM-T Easy kit (Promega, www.promega.com) following manufacturer's instructions. Plasmid DNA from single *E. coli* clones was isolated and sequenced while inserted within the pGEM-T Easy plasmid vector by the Genomics Core Research Facility at the University of Nebraska using a Beckman-Coulter CEQ8000-8-capillary DNA sequencer using dye-terminator chemistry. Sequencing was performed in both directions.

The 5′ end of the NADP^+^-IDH gene was amplified from *G. firmus* RNA using the 5′ RACE System for Rapid Amplification of cDNA Ends, Version 2.0, following the manufacturer's instructions (Invitrogen). The reverse primer for first-strand cDNA synthesis was CCGAGTCACATTATTCAT (bp 1227– 1244, Figure 1). The synthesized NADP^+^-IDH cDNA template was then used to amplify the 5′ end using the following gene-specific reverse primer: CTCCTTCAGACTTCTTGGCATAGGCA (bp 845–870) (Invitrogen), and the 5′ RACE Abridged Anchor Primer that was provided with the 5′ RACE kit. The PCR reaction (50µl) mixture contained the following: 35.5 µl DEPC-treated water, 5 µl PCR buffer (200 mM Tris-HCl, pH 8.4; 500 mM KCl; Invitrogen), 1 µl 10 mM dNTP mixture (Invitrogen), 3 µl 25 mM MgCl_2_ (Invitrogen), 2 µl 1.5mM, forward primer, 2 µl 0.2uM, reverse primer, 0.5 µl, *Taq* DNA polymerase ( 5U/µl, Invitrogen), and 1 µl template cDNA (1 µg/ml)). PCR amplification was performed as follows: 94° C for 2 minutes; 35 cycles at 94° C for 60 seconds, 55° C for 30 seconds; and 72° C for 90 seconds, followed by 72° C for 5 minutes, and 22° C for 10 seconds. Following PCR amplification, 10 µl of each reaction was analyzed on a 2% agarose TAE gel. A band of expected size (ca. 900 bp) was gel purified, extracted, reamplified under the same conditions, cloned using the pGEM-T Easy vector, and sequenced as described above.

The 3′ end of the NADP^+^-IDH gene was amplified from *G. firmis* RNA using the 3′-Full RACE Core Set according to the manufacturer's instruction (Takara Mirus Bio Inc., www.takara-bio.com). Reverse transcription of cDNA from RNA was achieved using the primer Oligo dT-3 sites Adaptor Primer that was provided in the kit. NADP^+^-IDH -specific forward primers (below) were used with the 3-sites Adaptor Primer to amplify NADP^+^-IDH cDNA products by hemi-nested PCR. The PCR reaction (50µl) contained the following: 36.5 µl distilled water, 5 µl PCR buffer (see above), 1 µl 10 mM dNTP mixture (Invitrogen), 1.5 µl DMSO, 2.5 µl 50 mM, MgCl_2_ (Invitrogen), 0.5 µl 10 µM forward primer, 1 µl 10 µM reverse primer, 1 µl *Taq* DNA Polymerase (5U/µl, Invitrogen), and 1 µl template cDNA (1ug/ml)). The first PCR amplification using the NADP^+^-IDH -specific primer AGCACCAAGAACACGATTCTGAAG (bp 7008–731, Figure 1) was performed as follows: 94° C for 2 minutes; 35 cycles of 94° C for 30 seconds, 50° C for 30 seconds, and 72° C for 90 seconds; followed by 72° C for 2 minutes and 22° C for 10 seconds. The second of the two hemi-nested PCR amplifications was performed using the same reaction components and PCR conditions as listed above for the first amplification except that the NADP^+^-IDH -specific primer was TGCCTATGCCAAGAAGTCTG (bp 845864), and the template consisted of 1 ul extract of gel-purified reaction mixture of the first PCR run. Following the second PCR amplification, 10µl of each reaction was run on a 2% agarose TAE gel. A single high intensity band of about 1 kb was observed. The rest of the reaction mixture was purified using a QIAgen PCR purification kit, the PCR product was ligated into a pGEM-T EASY vector, cloned, and sequenced as described above.

### Enzyme assay

*Gryllus firmus* NADP^+^-IDH activity was measured in 4–6 day-old adults using the following assay (Zhao and Zera 2001): 6.0 mM DL-isocitric acid trisodium salt, 4 mM MgCl_2_, 0.4 mM NADP^+^, in 50 mM MOPS (3[N-Morpholino]propanesulfonic acid) buffer, pH 8.0. Diluted enzyme was added to the assay cocktail (1 ml total volume) and incubated at 28° C in a temperature-regulated spectrophotometer for two minutes. After this time, production of NADPH was measured at 340 nm over a 2–6 min period. Controls involved measurement of activity in the absence of Na^+^-isocitrate. Background studies established that reaction rates during this time were initial rates (i.e. no measurable decrease in rates during the assay period), and were linearly related to enzyme concentration. Protein was measured using the Bradford assay using bovine serum albumen as a standard (Stoschek 1990).

### Enzyme specific activity in various organs and subcellular location

To determine the extent to which NADP^+^-IDH occurs in the mitochondrial vs. nonmitochondrial portions of the cell, fat body was homogenized 1:3 (w/v) in 10 mM phosphate buffer, pH 8.0 containing 0.5 mM EDTA, 10 mM Na^+^-azide, and 250 mM sucrose. Homogenate was centrifuged at 600 × g for 10 min to pellet cellular debris, and a small portion of the supernatant was saved. The remaining supernatant (300 µl) was centrifuged at 14,000 × g to pellet mitochondria, after which the supernatant was assayed for NADP^+^-IDH activity. The mitochondrial pellet was resuspended in 300 µl homogenization buffer, sonicated for a few seconds, and assayed for enzyme activity. Background studies showed that under these conditions, 84% of the activity of the mitochondrial marker enzyme succinate dehydrogenase occurred in the mitochondrial pellet while 95% of the cytoplasmic marker enzyme 6-phosphogluconate dehydrogenase occurred in the supernatant. Background studies also showed that resuspension and washing of the mitochondrial pellet resulted in a significant increase in succinate dehydrogenase activity in the supernatant indicating disruption of the mitochondria. Thus, this washing step was omitted.

### Enzyme purification

All purification steps were conducted in a cold room (4–7° C), or on ice. Frozen *G. firmus* (30 g; approximately equal number of LW and SW individuals, 4–6 days post adult eclosion) were chopped into small pieces, and were homogenized for about 30 sec using a Polytron PT10–35 homogenizer (Brinkman Instruments, www.brinkmann.com) in 150 mL of 50mM potassium phosphate buffer, pH 7.8, containing 0.1% β-mercaptethanol (βME), 5 mM EDTA, 0.5 mM phenylmethanesulfonyl fluoride (PMSF), and 10 µM of the protease inhibitor E64 (L-trans-carboxiran-2-carbonyl-L-leucylagmatine). Buffer pH dropped from 7.8 to 7.4 during homogenization. The homogenate was spun at 12,000 × *g* for 20 min, after which the top layer of fat was removed with a spatula, and the supernatant (referred to as “crude-homogenate”) was poured through four layers of cheesecloth. An equal volume of 40 percent polyethylene glycol (PEG; MW = 8,000) in homogenization buffer, but without PMSF or E-64, and at pH 7.4, was added slowly with stirring to the crude homogenate on ice. The solution was stirred for an additional 30 min after which it was spun at 12,000 × *g* for 30 min. The supernatant was removed and used immediately or was frozen at -20° C (90% retention of activity over a 10 day period when frozen; see Results).

The post-PEG supernatant was diluted 1:2 with distilled water to reduce its viscosity, and was passed through an 18 mL Cibacron Blue affinity column (Sigma; 1.6 cm i.d. × 9 cm) at a flow rate of 15 mL/h. The column had been equilibrated with 25 mM potassium-phosphate buffer pH 7.5, containing 0.05 % β-ME and 2.5 mM EDTA (= equlibration buffer A). Prior studies had demonstrated that the enzyme does not bind to the Cibacron Blue resin in the absence of Na-isocitrate (IC) and MgCl_2_. The eluant, plus an additional 40 ml equilibration buffer A wash, was brought to 1.2 mM IC and 4 mM MgCl_2_, and was passed through a new Cibacron Blue column (0.8 cm id × 5 cm; 2.5 ml) at 15 ml/h. This column had been equilibrated with equilibration buffer A, plus 1.2 mM IC and 4 mM MgCl_2_. The column was washed with 60 ml equilibration buffer A without IC or MgCl_2_. Greater than 90% of the loaded NADP^+^-IDH activity was retained on the column. NADP^+^-IDH was eluted with a 50 mL 0–0.5 mM NADP^+^ linear gradient at a flow rate of 30 ml/h. NADP^+^-IDH activity was found as a single peak in the first 25% of the gradient. Fractions containing > 5% of the total eluted NADP^+^-IDH activity (fractions 4 and 5 of the 20 2.5 ml fractions) were combined and were brought to 1.2 mM MgCl_2_ and 1.2 mM IC and were kept overnight on ice with little loss in activity. The post Cibacron Blue eluant was brought to 4 M NaCl, and the solution was loaded at 30 ml/h onto a 2.5 ml phenyl sepharose hydrophobic interaction column (Sigma, www.sigmaaldrich.com) 0.8 cm id, 5 cm height) that had been equilibrated with 25 mM potassium phosphate buffer, pH 7.5 containing 0.05% β ME, 5 mM EDTA (= equilibration buffer B), and 4 M NaCl. The column was washed with 25 mL of equilibration buffer B, followed by 70 ml of a 4 to 0 M linear NaCl gradient in equilibration buffer B. Twenty 3.5 ml fractions were collected. The column was washed with 14 ml buffer B and 4 additional fractions (3.5 ml) were collected. The enzyme eluted as a single broad peak beginning at the middle of the gradient and continuing through the additional buffer B wash. The five groups of fractions (12–15, 16–20, 21, 22, 23–24) that each contained > 8% of total eluted activity were separately concentrated to 1–2 ml using a Centricon 50 filter (Millipore, www.waters.com), spun at 5,000 × *g* for 20 min at 4° C, and subjected to SDS-PAGE. Fractions 22, and 23–24, which were judged to be homogeneously pure NADP^+^-IDH by SDSPAGE, were combined, brought to 30–40% glycerol, and used for kinetic analyses or frozen at -80° C.

### SDS and native polyacrylamide-gelelectrophoresis (PAGE)

Samples from various stages of enzyme purification were analyzed using standard SDS-PAGE electrophoresis (Garfin 1990) 8% T separating gel run for 3.5 h at 30 mA, constant current. Protein bands were visualized either by silver staining or with Coomassie Brillant Blue R 250 (Garfin 1990). Subunit molecular weights were estimated by linear regression analysis using Sigma molecular-weight standards (# M2789; myosin 205 kD, β-galactosidase 116 kD, Phosphorylase B 97.4 kD, bovine serum albumin 66 kD, ovalbumin 45 kD, carbonic anhydrase 29 kD, and soybean trypsin inhibitor 20 kD). Various organ homogenates were also subjected to native PAGE (7.5% T precast BIO-RAD (www.bio-rad.com) Ready Gels, Tris-HCl buffer, pH 8.2) to determine whether electrophoretically-distinct isoforms exist for this enzyme. Fat body homogenates were run using the same buffer system as that used in SDS PAGE, but without SDS. Gels used for other organ homogenates were prerun at 100 V for 2 h with a 20 mM Triscitrate, pH 8.2 tank buffer. Samples were then run on the same gels with 200 mM, pH 8.2 tank buffer for 4 h at 100 V. All native gels were run in a cold room at 4–7° C. Enzyme bands were visualized on native gels by staining for NADP^+^-IDH activity as in Murphy et al. (1996). Gels were incubated for 20 min in staining solution before adding phenazine methosulfate.

### N-terminal amino-acid sequence

Combined concentrated fractions of the last step in the purification protocol and concentrated eluant from the Cibacron Blue column (penultimate step in the purification protocol) were subjected to standard SDS PAGE, followed by transfer of proteins to a PVDF membrane using a Bio-Rad Mini Trans-Blot electrophoretic transfer cell. The transfer buffer contained 90% 10 mM 3[cyclohexylamino]-1 -propane-sulfonic acid, adjusted to pH 11.0 with NaOH and 10% methanol. Protein transfer was performed at constant 400 mA (about 65 volts) for 35 min with stirring at 4–7° C. The membrane was lightly stained with 0.25% Coomassie Blue R250 dissolved in 40% aqueous methanol and destained with 50% aqueous methanol. The presumptive NADP^+^-IDH band in the lane containing sample from the final purification step (only one band was observed on the membrane for this lane; see Results) was excised and amino-acid sequence determined by the Protein Structure Core Facility, University of Nebraska Medical Center using automated Edman degradation on an ABI Procise 494 sequencer. In a similar manner, the major non-NADP^+^-IDH bands from SDSPAGE of eluant from the Cibacron Blue affinity column were also excised and sequenced to identify contaminating proteins present in the latter stages of purification.

### Kinetic analysis

Michaelis constants (K_M_) for Na^+^-isocitrate (IC) and NADP^+^ were estimated for purified NADP^+^-IDH using the standard steady-state approach of measuring initial reaction rates as a function of substrate concentrations that bracketed the expected K_M_ (Fromm 1975). The standard NADP^+^-IDH assay was used except that either NADP^+^ or IC was varied, with the other substrate concentration being held constant at a saturating level (estimated in preliminary studies; see below). Preliminary studies demonstrated that these saturating substrate concentrations did not cause substrate inhibition. Assays were measured in triplicate at 28°, pH 8.0, the pH at which NADP^+^-IDH activities have typically been measured in *G. firmus* (Zhao and Zera 2001; Zera and Zhao 2003). In addition, K_M_s were measured at physiological pH 7.0 at the standard assay temperature of 28° C (Somero, 1981). At pH 8.0, IC concentrations of 1200, 100, 50, 30, 20, and 10 µM, were used with a fixed NADP^+^ concentration of 400 µM. NADP^+^ concentrations of 400, 100, 25, 10, 3, and 1 µM were used at a fixed IC concentration of 1200 µM. Rates in which IC was varied at 400 µM NADP^+^ were measured using a temperature-controlled Cary 50 (Varian, www.varianinc.com) spectrophotometer, while assays in which NADP^+^ was 100 µM or lower were measured using a temperature-regulated FLUO star Omega spectrofluorometer (BMG) at 28° C (excitation and emission wavelengths = 460 and 340 nm respectively). Less than 10% of substrate was consumed during the assay period to conform to steady-state requirements (Fromm 1975).

For K_M_ measurements conducted at pH 7.0, the two-substrate method of Duggleby (1979), was used: one set of six-eight replicate assays of the varied substrate was conducted at a substrate concentration near the K_M_, while a second set was conducted at a substrate concentration that gave rates that were near V_max_, with the fixed substrate also at a concentration that gave rates close to V_max_ (i.e. 20 µM or 600 µM IC at a fixed cone of 400 µM NADP^+^; 3 µM or 100 µM NADP^+^ with a fixed cone, of 1200 µM IC). This approach has the advantage of being much less laborious than the standard approach of measuring rates at five or more concentrations of the varied substrate (Duggleby 1979). It requires prior demonstration that the enzyme under study exhibits Michaelis-Menten kinetics, and a prior estimate of the K_M_, both of which were obtained in studies detailed in the preceding paragraph. Because the apparent K_M_S were estimated at the concentration of the fixed substrate that was essentially saturating (> 80 K_M_), they closely approximate (>98%) the true Michaelis constant. In control enzyme assays in which either Na^+^-isocitrate or NADP^+^ was not present in the assay mixture, no observable rates were observed. Kinetic constants were estimated from non-linear regression of the untransformed rate data using the program Enzfitter (Version 2.0, Biosoft, www.biosoft.com).

**Figure 2.**
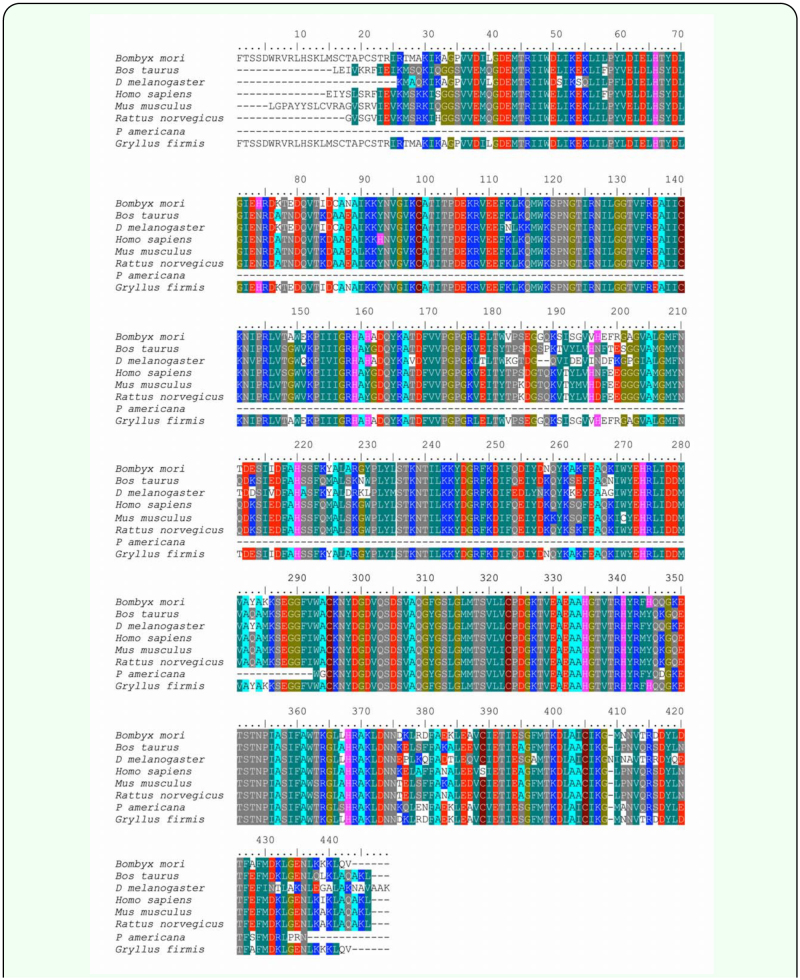
Results of ClustalW alignment of translated amino acid sequences of NAPD^+^-IDH for *Gryllus firmus* obtained in the present study and representative NADP^+^-IDH sequences for various insect and vertebrates. Sequences of the three vertebrate and three insect species used for alignment were also used to generate PCR primers for the initial amplification of the **381** bp internal sequence (see Methods). High quality figures are available online.

## Results

### Amplification and sequencing of the *Nadp^+^Idh* gene from *G. firmus*

The nucleotide sequence of the 381 bp amplicon (bp 708–1088, Figure 1), initially produced by PCR using *G. firmus* genomic DNA as template and degenerate primers constructed from conserved nucleotide sequences from vetebrate and insect NADP^+^-IDHS, exhibited strong similarity to published nucleotide sequences of mammalian and insect NADP^+^-IDH genes. Amplification of the 3′ and 5′ ends of the NADP^+^-IDH gene by RACE produced amplicons (bp 1–731 and bp 845–1755), which, when combined with the 381 bp internal sequence, yielded a 1755 bp amplicon that contained two potential ATG start codons at the 5′ end (bp 45–47 and 84–86; Fig. 1), multiple stop codons, 5′ and 3′ untranslated regions, and a polyadenylation tail (Figure 1). The downstream ATG start codon at nucleotide positions 84–86 (Figure 1) and adjacent nucleotides were identical to the canonical mRNA ‘Kozak sequence’, ACCAUGG (see Discussion), while the more upstream ATG (bp 45–47, Figure 1) was not embedded in this canonical sequence. Thus the more downstream ATG is identified as the preferred start codon (see Discussion).

**Figure 3.**
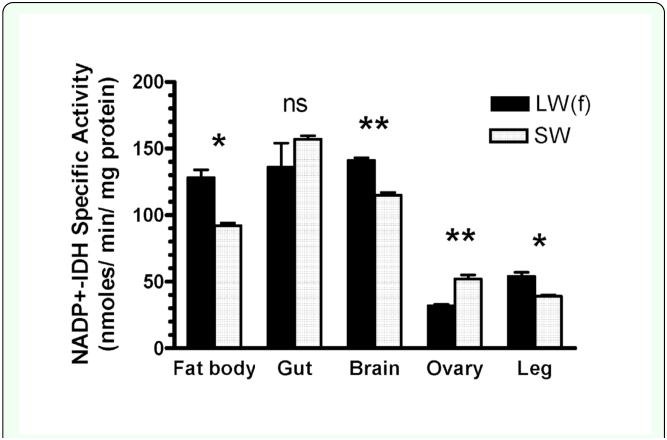
Specific activities (means±SEM) of NADP^+^-isocitrate dehydrogenase from various organs of long-winged, flight capable [LW(f)], or short-winged, flightless (SW) female *Gryllus firmus*. Symbols above histograms indicate results of t-tests comparing activities between LW(f) and SW females. Each mean was the average of two individuals. High quality figures are available online.

BLAST analysis indicated a high degree of similarity between the 1755 bp *G. firmus* NADP^+^-IDH nucleotide sequence, and NADP^+^-IDH sequences from four mammals (43–57%) and two insects, *B. mori* and *D. melanogaster* (51–58%). Aligned, translated amino acid sequences of NADP^+^-IDH from *G. firmus* and representative insects and vertebrates are presented in Figure 2. BLAST analysis also identified a high similarity between the translated amino acid sequence of *G. firmus* NADP^+^-IDH with corresponding sequences of mammals (72–75%) and *D. melanogaster* and *B. mori* (74–80%). These data collectively provide strong evidence that the amplified gene from *G. firmus* encodes an NADP^+^-IDH. This sequence has been deposited in GenBank (GenBank accession no. DQ886272).

### Stability studies of the NADP^+^-IDH

Prior to purification, experiments were conducted to determine the stability of NADP^+^-IDH under various conditions. Crude homogenate (post 12,000 × *g* supernatant in 50 mM K^+^-phosphate buffer, pH 7.4–7.9, containing 5 mM EDTA and 0.1% βME) retained 65% NADP^+^-IDH activity over 6 days when kept on ice. Stability of the enzyme was enhanced (80% activity retained after 6 days on ice) by the addition of glycerol or PEG (15–20% v/v), 10 µM NADP^+^, and 1 mM PMSF. When kept frozen at -20° C, 100%, 90%, and 70% activity was retained after 1 day, 10 days, and 6 months, respectively. Stability of frozen enzyme allowed stockpiling of homogenates of crickets of a specific age range for further purification.

### NADP^+^-IDH specific activities in various tissues, subcellular location, and native gel electrophoresis

High NADP^+^-IDH specific activity was found in crude homogenates of fat body, gut, and head, while much lower specific activity was observed in the ovary and leg (Figure 3). Differential centrifugation demonstrated that the majority (858 ± 8 nmol/min; 90.1%) of fat body NADP^+^-IDH activity occurs in the post 14,000 × g supernatant vs. the resuspended mitochondrial pellet (94±1 nmoles/min; 9.9%) and thus is largely, if not exclusively, nonmitochondrial. Similar results were obtained for whole-cricket homogenates (data not shown). Native PAGE electrophoresis of fat body homogenate from 20 *G. firmus* using the standard Laemmli PAGE Tris-glycine/Tris-HCl buffer system (Garfin 1990), but without SDS, revealed a single NADP^+^-IDH band of similar mobility in each individual (see Figure 3A for a representative gel). When this buffer system was used, no bands were observed on native PAGE or agarose gels of homogenates of organs other than fat body (e.g. gut, head, leg, ovaries) even though several of these organs exhibited specific activities that were equivalent to that of fat body (Figure 3). A single band of roughly similar mobility was seen for NADP^+^-IDH from fat body, head, and leg when homogenates were run in Triscitrate pH 8.2 PAGE gels (see Methods), although the resolution was not as good as that for the Tris-glycine/Tris-HCl gels. No band of NADP^+^-IDH activity was observed for gut homogenate under any native electrophoretic conditions.

**Table 1.**
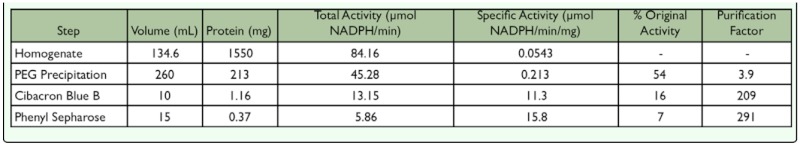
Purification of NADP^+^-lsocitrate dehydrogenase from *G. firmus*

### NADP^+^-IDH protein purification

The purification protocol used in the present study resulted in an approximately 300-fold increase in specific activity of NADP^+^-IDH with a 7% yield of homogeneously pure enzyme (Table 1). Specific activity of the purified NADP^+^-IDH was 15.8 µmol NADPH/min/mg protein (Table 1). The key step in the purification protocol was substrate-dependent Cibacron-Blue affinity chromatgraphy. This procedure allowed NADP^+^-IDH to pass through the Cibacron-Blue affinity column when Na+-isocitrate (IC) and MgCl_2_ were absent from the buffer, a step which removed a variety of contaminating proteins, including several dehydrogenases and kinases. NADP^+^-IDH could then be bound to the same affinity resin (different column) when IC and MgCl_2_ were present in the buffer, and subsequently eluted with an NADP^+^ gradient. This single step resulted in a greater than 50–100-fold increase in specific activity. The subsequent hydrophobicinteraction chromatographic (HIC) step removed the few remaining impurities, the most prominent of which was identified as glyceraldehyde-3-phosphate dehydrogenase, based on the similarity of its 35 kD subunit molecular mass, and N-terminal sequence (AKIGINGFGRIGDLVLRAAI) with other G-3-PDHs (BLAST analysis). G-3-PDH coeluted during the first half of the NADP^+^-IDH activity peak, while the second half exhibited a single band (46.3±1.1 kD) on SDS-PAGE gels that were either Coomassie or silverstained (Figure 5A, B). This molecular mass estimate was derived from regression analysis of the relative mobility of molecular mass markers on five separate SDS-PAGE gels from five different purifications (range of estimates: 43.8–49.8). In two separate preparations, each starting with 30 g. of frozen *G. firmus*, 240 µg or 370 µg of homogeneously purified NADP^+^-IDH was obtained.

**Figure 4.**
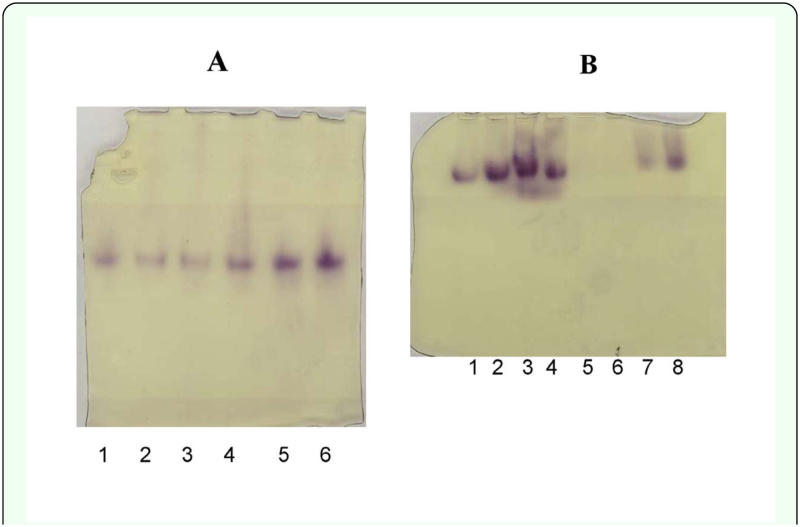
Native PAGE gels (7.5% T separating gel) stained for NADP^+^-IDH. Panel A: Representative gel showing a single NADP^+^-IDH band of the same mobility in each of 6 fat body homogenates of *Gryllus firmus*. Buffer components were the same as those in the SDS gels, but without SDS. An additional 14 individuals exhibited the same single band. Panel B: A 7.5% T gel showing a single NADP^+^-IDH band of indistinguishable mobility in various organ homogenates of *G. firmus*: lanes 1, 3, 5, 7 were homogenates from one individual and lanes 2, 4, 6, 8 were homogenates from another individual; lanes I and 2 = fat body, lanes 3 and 4 = head, lanes 5 and 6 = gut, lanes 7 and 8 = leg. As described in the Materials and Methods, the gel was pre-run and run with a Tris-citrate pH 8.2 tank buffer. No bands were observed in the gut homogenates (lanes 5 and 6). High quality figures are available online.

**Table 2.**
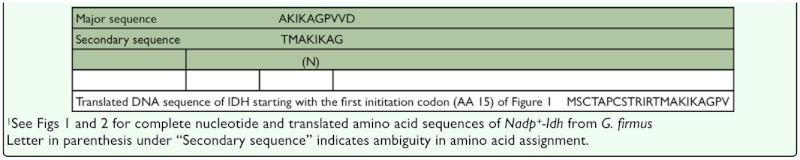
N-terminal ammo-acid sequences of the two NADP^+^-dependent isocitrate dehydrogenase enzyme variants purified from *Gryllus firmus*

### N-terminal sequencing and IDH aminoacid sequence analysis

N-terminal sequencing of the single 46.3 kD band excised from the SDS-PAGE gel of eluant from the final purification step indicated the presence of two NADP^+^-IDH proteins, termed IDH-1 and IDH-2(tr) (Table 2), with NADP^+^-IDH-1 being about 1.7 times as abundant as the other protein. N-terminal amino-acid sequences (8–10 amino acids) of both proteins matched the translated nucleotide sequence of the *Gryllus firmus* NADP^+^-IDH gene that was amplified in the present study (Table 2 and Figure 1; see below), and the sequences were very similar to each other. These results are consistent with two NADP^+^-IDH proteins being transcribed from different start codons of the same NADP^+^-IDH gene, with NADP^+^-IDH-2(tr) being a truncated (proteolytically-cleaved) protein transcribed from the more upstream start codon (see Discussion). These results are also consistence with multiple NADP^+^-IDH proteins being encoded by different genes.

**Figure 5.**
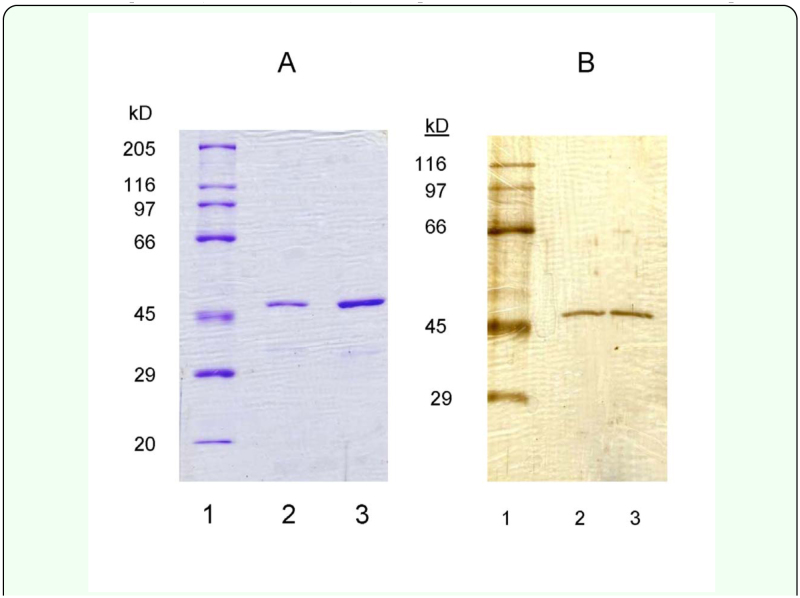
SDS PAGE gels (separating gels: 8% T) of NADP^+^-isocitrate dehydrogenase, obtained from the final purification step of two different purification runs, were stained with Coomassie Blue (Panel A; 10 µg and 40 µg of enzyme per well of lanes 2 and 3) or were silver stained (Panel B; 0.5 µg enzyme per well of lanes 2 and 3). See Methods for identity of the molecular mass markers. High quality figures are available online.

**Figure 6.**
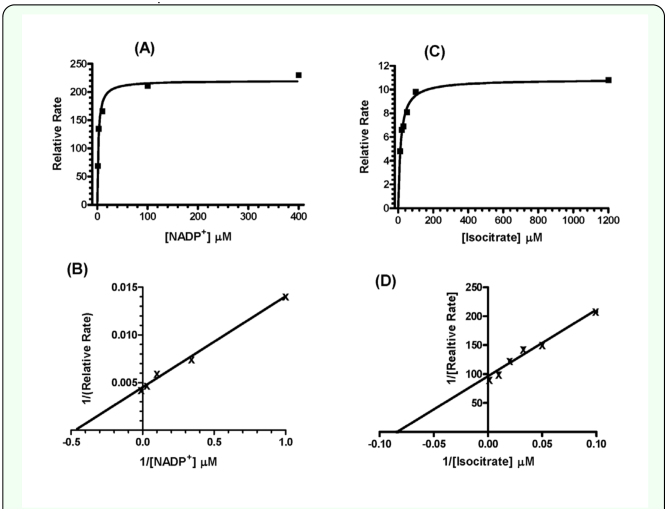
Graphical results of kinetic analysis of purified NADP+-isocitrate dehydrogenase. Non-linear regession lines of untransformed initial rates (top panels), and linear regession of reciprocal rates (Lineweaver-Burk analysis) were performed using Enzfitter (Version 2.0; © Biosoft, 1997). High quality figures are available online.

### Sequence analyses for subcellular location and enzymatic properties

Amino-acid sequence analysis by PSORT II (Horton and Nakai 1997) indicated that the more abundant NADP-IDH-1 has a much greater probability of being located in the cytoplasm (61%) rather than mitochondrion (4%). The same is true for NADP^+^-IDH-2(tr) (44% probability of being cytoplasmic vs. 13% mitochondrial). Similar results regarding the likely cytoplasmic location of NADP^+^-IDH-1 and NADP^+^-IDH-2(tr) was obtained by WoLF PSORT (Horton et al. 2006) and TargetP (Emanuelsson 2007). No C-terminal peroxisomal targeting sequence, nor any other organelle targeting sequence was found in either IDH sequence except for an endoplasmic reticulum membrane retention signal (“KKLQ” motif), which occurs at the C-terminus of NADP-IDH-1 (identified by PSORT II; Figure 1). The absence of a peroxisomal targeting signal supports a cytoplasmic location of the enzyme. Molecular mass of NADP^+^-IDH-1, estimated from ProtParam (Gasteiger et al., 2005) as 46.2 kD, is close to the molecular mass of the purified NADP^+^IDH measured on the SDS PAGE gel (46.3 kD, see above). Isoelectric points, estimated from the translated amino acid sequence by ProtParam, were 7.3 for NADP^+^IDH-1, and 8.0 for NADP^+^IDH-2(tr). Attempts to measure isoelectric points experimentally using enzyme at various stages of purity were unsuccessful

### Enzyme kinetics

Purified NADP^+^-IDH exhibited standard Michaelis-Menten kinetics for the varied substrate [Na^+^-isocitric acid (IC) or NADP^+^] in the presence of an essentially-saturating level (> 80 K_M_) of the fixed, alternate substrate at pH 8.0 (Figure 6). Untransformed rate data exhibited a good fit to a curve (rectangular hyperbola) derived from the Michaelis-Menten equation (Figure 6A, C,). Lineweaver-Burk plots (Figure 6B, D) of the inverse transformed rate data exhibited a good fit to a straight line, also consistent with the NADP^+^-IDH exhibiting simple Michaelis-Menten kinetics. At pH 8.0 (28° C), the K_M(Ic)_ was 14.7 ± 1.8 µM, while the K_M(NADP+)_ was 2.3 ± 0.4 µM. Kinetic constants, estimated at pH 7.0 (28° C), by the method of Duggleby (1979), were very similar to those measured at pH 8.0: K_M(IC)_ = 13.1 ± 1.9 µM, K_M(NADP+)_ = 4.3±0.48 µM.

## Discussion

### NADP^+^-IDH enzyme and *Nadp^+^-Idh* gene from *G. firmus:* structural and functional properties and comparison with NADP^+^-IDH enzymes and genes from other organisms

Several lines of evidence indicate that (1) the protein purified in the present study from whole-body homogenate of *G. firmus* was cytoplasmic NADP^+^-IDH, consisting of two very similar variants of indistinguishable molecular mass, and (2) the amplified gene is NADP^+^-IDH, which encodes the purified NADP^+^-IDH enzyme. The N-terminal amino acid sequences and subunit molecular mass of the purified proteins matched the N-terminal amino acid sequences and molecular mass deduced from the NADP^+^-IDH amplified gene. Both the nucleotide and deduced aminoacid sequences of the NADP^+^-IDH gene from *G. firmus* exhibited a high degree of identity with nucleotide and amino-acid sequences of NADP^+^-IDH proteins of mammals and insects (Blast Analysis, Results, Figure 2). Finally, the purified NADP^+^-IDH exhibited kinetic properties similar to homogeneously-purified NADP^+^-IDH proteins from a variety of other organisms (see below).

The subunit molecular mass of NADP^+^-IDH from *G. firmus* (46.3 kD) is similar to that reported for NADP^+^-IDH proteins isolated from the silkworm, *Bombyx mori* (44 kD; Miake et al. 1977a), and numerous other animals [values typically in the range of 45–50 kD; summarized in Head 1980], and yeast (Lofrus et al. 1994). Although the native molecular mass was not determined, NADP^+^-IDH in *G. firmus* probably exists as a dimer composed of equal subunits, as is the case for virtually all other eukaryotic NADP^+^-IDHs (Head 1980). The K_M_s for Na^+^-isocitrate (13–14 µM) and NADP^+^ (2–4 µM) for purified NADP^+^-IDH from *G. firmus* (Results) were similar to values reported for purified NADP^+^-IDHs from *Drosophila melanogaster* and *B. mori* (K_M_s for IC ranged from 4–24 µM; K_M_s for NADP^+^ ranged from 4–15 µM; Williamson et al. 1980 Bentley et al. 1983; Miake et al. 1977a), and vertebrates (e.g. Balamir 1983; Gonzalez-Villaseñor and Powers 1985; Contreras-Shannon and McAlister-Henn 2004). Specific activity (15.8 µmol/min/mg protein) of the purified protein from *G. firmus* was similar to that of NADP^+^-IDH purified from *D. melanogaster* (19.7 µmol/min/mg protein; Williamson et al. 1980) and was about half that of the enzyme from *Bombyx* (26.3 µmol/min/mg protein; Miake et al. 1977a).

The *G. firmus* NADP^+^-IDH amino acid sequence contained the highly conserved “isocitrate and isopropylmalanate signature sequence” (NYDGDVQSDSVAQGFGSLG; AA 891–942, Figure 1; AA 297–315, Figure 2) found in all cytoplasmic and mitochondrial NADP^+^-IDH sequences of eukaryotes (Nekrutenko et al. 1998). We obtained the NADP^+^-IDH nucleotide sequence of *G. firmus* using genomic *DNA* for the internal portion of the gene (the initial 381 bp amplicon; bp 708– 1088, Fig. 1), and cDNA for the rest of the sequence (3′ and 5′ RACE). Subsequently, the entire coding sequence of this gene was reamplified exclusively using genomic DNA from each of several different *G. firmus* females (see below), and the 381 bp internal sequence was reamplified using cDNA (S. Neuman, R. Schilder, M. Hoidal, A. J. Zera, unpubl). Nucleotide sequences were identical to those obtained in the present study except for a few nucleotide substitutions (see below). Thus, the NADP^+^-IDH gene from *G. firmus* contains no introns.

### Nature and significance of IDH variants in *G. firmus*

An unexpected finding of the present study was the existence of two NADP^+^-IDH variants from *G. firmus*. Although purified NADP^+^-IDH protein exhibited a single band of 46.3 kD on SDS-PAGE (Figure 5), amino-acid sequencing revealed two proteins whose sequences differed at the N-terminus (Table 2). Because the N-terminal sequences of both proteins matched the translated amino-acid sequence of the *Nadp^+^-Idh* gene (Table 2, Figure 1) and the molecular masses of these proteins were indistinguishable on SDSPAGE, they appear to be NADP^+^-IDH variants of very similar sequence, possibly differing by only two N-terminal amino acids. The absence of deviations from simple Michaelis-Menten kinetics for purified NADP^+^-IDH (Figure 6), which was a mixture of the two NADP^+^-IDH variants, indicates no measurable differences in kinetic constants between the variants, further attesting to their similarity. Finally, only one NADP^+^-IDH band was observed in native PAGE gels (Figure 4).

The existence of two different N-terminal sequences, both of which match the NADP^+^-IDH gene sequence of *G. firmus*, combined with the existence of two potential ATG initiation sites in the NADP^+^-IDH nucleotide sequence, suggest that the two NADP^+^-IDH enzymes may be encoded by one gene in *G. firmus*. The NADP^+^-IDH-1 (more abundant; see Results) amino acid sequence is consistent with a protein whose initiation sequence is the second ATG (bp 84–96; Figure 1). The second (lesser abundant) NADP^+^-IDH (designated NADP^+^-IDH-2(tr)) is consistent with an enzyme (NADP^+^-IDH-2) transcribed from the first ATG (bp 45–47, Figure 1). Subsequent cleavage of an Arg-Thr bond (denoted by the underlined RT sequence in Figure 1), which is a tryptic cleavage site, would produce a protein whose N-terminal sequence is the same as that of NADP^+^-IDH-2(tr), where “tr” designates a truncated protein due to proteolytic cleavage. If this indeed is the case, it is currently unknown whether the proteolytic cleavage represents a normal processing of the protein, or a degradation artifact produced during purification. An alternate possibility is that the two NADP^+^-IDH variants are products of different genes. Using genomic DNA, the entire NADP^+^-IDH coding sequence was reamplified through several hundred base pairs of 3′ non-coding sequence (bp 41–1541 of Figure 1) in each of 21 *G. firmus* derived from 3 LW and 3 SW genetic stocks (2–4 individuals per stock) (R Schilder, M Hoidal and AJ Zera, unpublished data). The sequences are very similar with only six nucleotide differences (5 synonymous) being observed among the 21 sequences in the coding region and no sequence variation in the 3′ non-coding region. Thus, if the NADP^+^-IDH variants are derived from different genes, the sequences of these genes are likely very similar.

The existence of multiple potential start sites is common in eukaryotic genes (Kochetov 2006; Wang and Rothnagel 2004). The “strength” of a potential mRNA start site for translation is determined by its context, that is, the specific nucleotides adjacent to the ATG sequence of the mRNA (Kozak 1994; Rogozin et al. 2001; Wang and Rothnagel 2004). An optimal start site is identified as one in which a purine occurs at position -3 and guanine occurs at position +4 (“A” of ATG = position 1) (Kochetov 2006). Some ribosomes do not recognize an mRNA AUG as the start codon if the ‘context’ is not optimal, and translation begins at the next AUG. The optimal start site ‘Kozak sequence, ACCAUGG’ (Kozak 1994; Rogozin et al. 2001), corresponds exactly to the sequence at bp 81–87 (Figure 1), while the more upstream ATG (bp 45–47, Figure 1) has neither of these preferred nucleotides at -3 or +4. Thus, the greater amount of NADP^+^-IDH-1 as opposed to NADP^+^-IDH-2(tr) found in the N-terminal sequencing is consistent the former being the preferentially translated NADP^+^-IDH.

The production of multiple NADP^+^-IDHs isozymes from different initiation sites of a single NADP^+^-IDH gene in *G. firmus*, while uncommon, would not be without precedent. Many plant, animal, and yeast species contain multiple NADP^+^-IDH isozymes that are differentially located in the cytoplasm, mitochondrion, or peroxisomes and which exhibit compartment-specific functions (Plaut et al. 1983; Gonzalez-Villaseñor and Powers 1985; Huh et al. 1993; Loftus et al. 1994; Nekrutenko et al. 1998; Szewczyk et al. 2001; Contreras-Shannon and McAlister-Henn 2004). In virtually all these cases, NADP^+^-IDH specific isoforms are encoded by different genes that have arisen evolutionarily by several successive rounds of gene duplication (Nekrutenko et al. 1998). However, a notable exception is the fungus, *Aspergillus nidulans*, which has two different NADP^+^-IDH isoforms that are targeted to different organelles, and which are produced from one-and-the-same gene using different initiation sites (Szewczyk et al. 2001).

Although multiple NADP^+^-IDH variants exist in *G. firmus*, results of differential centrifugation and bioinformatic analysis indicate that both NADP^+^-IDH variants are primarily, if not exclusively, located in the cytoplasm where they likely function primarily in fatty-acid biosynthesis. The high NADP^+^-IDH specific activity in fat body (Figure 3), the main site of lipogenesis, is consistent with this idea. The functional significance of the NADP^+^-IDH-1, NADP^+^-2(tr), or NADP-2 - the presumed parent of NADP-2(tr) - variants are unknown at present. On the one hand, these variants might be differentially expressed in various tissues, organs, or morphs (enzyme was purified from a mixture of LW and SW morphs) where they might exhibit different developmental or organ-specific functions. On the other hand, these variants may be functionally equivalent, and may simply reflect noise in gene expression. Additional studies will be required to address this topic. Because the NADP^+^-IDH gene sequences of *G. firmus* and the silkworm, *B. mori*, are very similar (Figure 2), with two potential start codons present in the gene of each species, it is likely that multiple NADP^+^-IDHs also exist in *B. mori*. Interestingly, and in contrast to *G. firmus, B. mori* clearly exhibits multiple electrophoretic forms of NADP^+^-IDH that are differentially expressed in various organs (Miake et al. 1977a). However, *B. mori* contains a separate mitochondrial NADP^+^-IDH gene (GenBank Accession No. DQ311190), which might contribute to the NADP^+^-IDH isozymic diversity in this species. In contrast, the *Drosophila* NADP^+^-IDH nucelotide sequence only contains one ATG intitiation codon in this region where two potential initiation codons occur in *G. firmus* (Figures 1, 2) and *B. mori* (Figure 2), and thus may produce only one mature protein from this gene.

Although considerable information is available on the physical and functional properties of NADP^+^-IDH in vertebrates and yeast (see above references), only rudimentary published information is available for this enzyme in insects. For example, even basic issues such as the number of Nadp^+^-Idh genes and proteins, the existence of organ-specific or subcellular isozymes, and even the subunit composition of the native enzyme are uncertain in insects, especially *Drosophila*. For example, using electrophoretic variants, different groups have mapped NADP^+^-IDH to two different locations on the third chromosome in *D. melanogaster* (summarized in Bentley et al. 1983). Williamson et al. (1980) also reported two NADP^+^-IDH subunits of different mass (50 kD and 60 kD) on SDS gel electrophoresis and suggested that the dimeric IDH may be composed of subunits derived from different loci. Bioinformatic analyses of rapidly accumulating *Drosophia* genome sequences will presumably allow resolution of some of these uncertainties.

### NADP^+^-IDH purification

Standard chromatographic protocols were employed to purify NADP^+^-IDH from *G*. *firmus*. The key step was dye-ligand affinity chromatography coupled with a prior PEG step to precipitate non-NADP^+^-IDH proteins, and a subsequent hydrophobic-interaction (HIC) step to remove the few remaining contaminating proteins. The 291 foldpurification of the enzyme from *G. firmus* (Table 1) was similar to that for NADP^+^-IDH purified from *Bombyx mori* (376-fold; Miake et al. 1977a) and considerably greater than that for the enzyme from *D. melanogaster* (94-fold; Williamson et al. 1980).

The requirement that Na^+^-isocitrate (IC) and MgCl_2_ be present in the buffer in order for NADP^+^-IDH from *G. firmus* to bind to the Cibacron Blue resin (see Methods) was unexpected. NADP^+^-IDHs have often been purified by affinity chromatography using immobilized dyes, which are thought to mimic NADP^+^; or by immobilyzed NADP^+^-analogues, or NADP^+^ itself, without the necessity of having IC-MgCl_2_ (the actual substrate of the enzyme) present in the buffer (e.g. Miake et al. 1977a; Williamson et al., 1980 Head, 1980; Gonzalez-Villaseñor and Powers 1985); Because NADP^+^-IDH exhibits a random sequential reaction mechanism (Northrup and Clelland 1974; Miake et al. 1977b; Farrell et al. 1993), the enzyme is expected to have the capability to bind to the nucleotide-mimicing Cibacron-Blue dye in the absence of substrate. Kinetic studies of mammalian NADP^+^-IDHs suggest a possible explanation for this puzzling observation. Farrell et al. (1993) reported that cytososlic NADP^+^- IDH from bovine mammary gland, binds IC-Mg^++^ outside of the active site resulting in a conformational change that influences the kinetics of the reaction (eliminates an initial lag phase). NADP^+^-IDH in *G. firmus* also exhibits an initial lag phase in the forward reaction with IC and NADP^+^, which is eliminated by pre-incubation of the enzyme with IC-MgCl_2_ (unpublished data). Thus, binding of IC-MgCl_2_ outside the active site of NADP^+^-IDH from *G. firmus*, may be required for this enzyme to attain a conformation necessary to bind to the Cibicron-Blue resin.

### Future studies

Purification of the NADP^+^-IDH protein and amplification of the corresponding NADP^+^-IDH gene has provided us with the tools necessary to investigate the molecular and biochemical causes of differences in NADP^+^-IDH activity between morphs of *G. firmus* (Zera 2005; Zera and Harshman 2011). These morphs are adapted for flight vs. reproduction and differ dramatically in the rate of triglyceride biosynthesis. In a companion study we report that morph differences in both NADP^+^-IDH enzyme protein concentration and transcript abundance, but not enzyme catalytic efficiency, or post-translational modification, account for differences in enzyme activity (R. Schilder and A. J. Zera, ms in prep.).

## Abbreviations:

**DMSO**,: dimethyl sulfoxide;
**EDTA**,: ethylenediaminetetraacetic acid;
**NADP^+^**,: nicotinamide adenine dinucleotide phosphate;
**NADPH**,: reduced nicotinamide adenine dinucleotide phosphate;
**PVDF**,: polyvinylidene fluoride
